# Population pharmacokinetics of mefloquine given as a 3-day artesunate–mefloquine in patients with acute uncomplicated *Plasmodium falciparum* malaria in a multidrug-resistant area along the Thai–Myanmar border

**DOI:** 10.1186/s12936-018-2466-3

**Published:** 2018-09-03

**Authors:** Richard M. Hoglund, Ronnatrai Ruengweerayut, Kesara Na-Bangchang

**Affiliations:** 1 Mahidol Oxford Tropical Medicine Research Unit, Faculty of Tropical Medicine, Mahidol University, Bangkok, Thailand; 2 Centre for Tropical Medicine and Global Health, Nuffield Department of Medicine, University of Oxford, Oxford, UK; 3 Mae Sot General Hospital, Mae Sot, Tak Thailand; 4 Center of Excellence in Pharmacology and Molecular Biology of Malaria and Cholangiocarcinoma, Thammasat University, Pathumtanee, Thailand

**Keywords:** Malaria, Population pharmacokinetics, Artemisinin-based combination therapy, Artesunate, Mefloquine

## Abstract

**Background:**

Low mefloquine exposure has been shown to contribute to treatment failure in patients with uncomplicated falciparum malaria following a 3-day artesunate–mefloquine combination. The present study aimed to develop a population pharmacokinetic model for mefloquine based on whole blood concentration–time profiles of this target population for further dose optimization.

**Methods:**

A total of 129 Burmese patients aged above 15 years who presented with typical symptoms of malaria and had a blood smear positive for *Plasmodium falciparum* were included in the study. All were treated with the standard 3-day combination regimen of artesunate and mefloquine consisting of mefloquine for 2 days and artesunate for 3 days. Blood samples were collected before and at different time points after drug administration from different sub-groups of patients. Mefloquine concentrations were quantified in whole blood using high-performance liquid chromatography. A non-linear mixed-effect modelling approach was applied for population pharmacokinetic analysis using the NONMEM v7.3 software. Covariates investigated (body weight, gender, admission parasitaemia, and molecular markers of mefloquine resistance) were investigated in a step-wise manner using the SCM functionality in Perl-Speaks-NONMEM.

**Results:**

Population pharmacokinetic analysis of mefloquine was performed in all patients with a total of 653 samples. Whole blood mefloquine concentration–time profiles were described by a two-compartment disposition model. Of the covariates investigated, none was found to have a significant impact on the pharmacokinetics of mefloquine. Significant differences in maximum concentration (C_max_) and elimination half-life (t_1/2_) were found in patients who had treatment failure (36 cases) compared to patients with successful treatment (107 cases).

**Conclusion:**

The study successfully describes the pharmacokinetics of mefloquine following a 2-day treatment of mefloquine as a part of a 3-day artesunate–mefloquine in patients with uncomplicated falciparum malaria from Thailand. A model has been developed which adequately describes the pharmacokinetics of mefloquine. More extensive clinical studies including both adults and children are needed to fully characterize the pharmacokinetics of mefloquine.

## Background

Malaria remains a significant infectious disease that kills over 1200 people every day [1]. The latest malaria report from the World Health Organization (WHO) shows that the number of malaria cases in 2017, which has previously been in steady decline since 2010, has now increased compared to the year before (with an estimated 216 million cases in 2016 compared to 211 million in 2015) [1, 2]. One of the main reasons for this resurgence is the development and spread of multi-drug-resistant *Plasmodium falciparum* [1]. To deal with the threat of resistance of *P. falciparum* to monotherapy, artemisinin-based combination therapy (ACT) was recommended by WHO in 2006 as a strategy to counteract the increasing resistance of *P. falciparum* to anti-malarials, as well as to prevent disease transmission and reduce the risk of drug resistance [3]. ACT consists of an artemisinin drug with a potent schizonticidal activity, together with a long elimination half-life partner drug, which is present in the blood at therapeutic concentrations for at least several of the parasite life cycles to prevent recrudescence [4]. These combinations have been shown to result in well-tolerated anti-malarial treatment, which acts rapidly and has a reliable and sustained efficacy. Their increasing use has been a critical factor behind the reduction in malaria transmission in Asia and other endemic areas of the world [4]. Unfortunately, artemisinin resistance has emerged during the last decade and has unfortunately spread within southeast Asia [5].

Among the recommended ACT regimens for falciparum malaria, artesunate–mefloquine combination was introduced as first-line treatment of multi-drug-resistant, uncomplicated falciparum malaria in Thailand before WHO recommendation. The combination n was initially used in 1995 as a 2-day combination regimen of 25 mg/kg body weight of mefloquine and 12 mg/kg body weight of artesunate to ensure better compliance [6]. The recommendation changed to a 3-day treatment in 2007 to follow WHO recommendation. Recently, the combination has been replaced by dihydroartemisinin–piperaquine due to the low clinical efficacy of artesunate–mefloquine combination [7]. The resistance of *P. falciparum* to ACT is of great concern to global malaria control programmes as alternative treatment options are limited.

Introduction of the triple combination of anti-malarials instead of the standard two-drug combination has been suggested as a further strategy to control artemisinin resistance [8]. Artesunate–mefloquine combination could be part of these triple combination or could replace dihydroartemisinin–piperaquine in areas with piperaquine resistance is prevalent. In some countries, such as Vietnam, mefloquine has been added to the current dihydroartemisinin–piperaquine as first-line treatment. Altogether, this information highlights the necessity and importance of gaining more knowledge of the pharmacokinetics of the partner drug mefloquine in target populations at risks. It is therefore important to adequately describe the pharmacokinetics of mefloquine in specific populations for appropriate dose optimization. In addition, the developed pharmacokinetic models can be further applied to predict optimal dose regimens in specific populations. The present study aimed to develop a population pharmacokinetic model for mefloquine based on data from adults with uncomplicated falciparum malaria in Thailand.

## Methods

### Patients and treatment

This population pharmacokinetic study was a part of the study protocol to investigate the clinical efficacy of a 3-day artesunate–mefloquine combination in patients with acute uncomplicated falciparum malaria in an endemic area along the Thai–Myanmar border. The study was conducted at Mae Tao clinic for migrant workers, Tak Province, Thailand, from March 2008 to February 2009. Ethical approval of the study protocol was granted by the Ethics Committee of the Ministry of Public Health of Thailand. Detailed information of the study procedures and results of clinical efficacy assessment, including the relationship with drug concentrations were previously described in detail [9].

Burmese patients, aged over 15 years, who had typical symptoms of malaria and were positive for *P. falciparum* diagnosed with blood smears, were included in the study. Inclusion criteria for enrolment in the study were patients with acute uncomplicated falciparum malaria according to the WHO protocol for areas with low to moderate malaria transmission [10]; patients with signs of severe or complicated malaria [11]; history of hypersensitivity reactions to any of the study drug; the presence of severe malnutrition; febrile diseases other than malaria. Pregnant or breast-feeding women were not included. All patients provided written informed consents before study participation.

All patients received a 3-day combination regimen of artesunate and mefloquine. An initial dose consisting of 4 mg/kg body weight of artesunate (200 mg, 4 tablets of 50 mg artesunate, Atlantic Pharmaceutical Company, Thailand) and 15 mg/kg body weight of mefloquine (750 mg, 3 tablets of 250 mg mefloquine; Atlantic Pharmaceutical Company, Thailand) was given on the first day of the study. On the second day, the patients received 4 mg/kg body weight of artesunate (200 mg, 4 tablets of 50 mg artesunate) and 10 mg/kg body weight of mefloquine (500 mg, 2 tablets of 250 mg mefloquine). On the third day, mefloquine was not administrated; instead artesunate (4 mg/kg body weight) was given together with 0.6 mg/kg body weight primaquine (2 tablets of 15 mg primaquine; Government Pharmaceutical Organization of Thailand). All doses were observed and patients were followed for 30 min. If patients vomited within 30 min of treatment, the dose was repeated. Anti-pyretic paracetamol and anti-emetic dimenhydrinate were administered if necessary. Patients were requested to return for follow-up on days 7, 14, 21, 28, and 42, or if the patient experienced fever or malaria symptoms. Parasites were counted (Giemsa-stain) at each visit, and general symptoms were recorded.

Genotyping was performed on paired samples of *P. falciparum* DNA to distinguish between re-infection and recrudescence [12]. Polymerase chain reaction (PCR) restriction fragment length polymorphism was used to detect mutations in the following molecular markers (coding for mefloquine resistance): *P. falciparum* multi-drug resistance 1 (*pfmdr 1*-N86Y, *pfmdr 1*-Y184F, *pfmdr 1*-S1034C, *pfmdr 1*-N1042D, and *pfmdr 1*-D12467), *P. falciparum* chloroquine resistance transporter (*pfcrt*-K67T, *pfcrt*-A220S, *pfcrt*-Q271E, *pfcrt*-N326S, *pfcrt*-I356T, and *pfcrt*-R371I), and *P. falciparum* ATPase 6 (*atp 6*-L263E, *atp 6*-E431K, *atp 6*-N569K, and *atp 6*-A623E) [13–15]. The *pfk13* sequencing was performed at K13-propeller domain (codons 440–680, 720 bp) [16]. The *pfmdr1* gene copy number was determined by SYBR Green I real-time PCR [17].

### Blood samples for pharmacokinetic study

Blood samples (1 ml of whole blood) were collected into sodium heparinized tubes before and after the first dose (at 1, 2, 6, 12, 24, 25, 36, 37, 48, and 49 h post-dose and on day 3, 7, 12, 14, 17, 21, 22, 23, 24, 27, 28, 32, 33, 35, and 42 post-dose) for determination of mefloquine concentrations. Collected samples were stored at − 20 °C until transported to Centre of Excellence in Pharmacology and Molecular Biology of Malaria and Cholangiocarcinoma, Chulabhorn International College of Medicine, Thammasat University. All samples were stored at − 80 °C until drug quantification.

### Drug quantification

Mefloquine concentrations were quantified using liquid chromatography according to a previously published method [18]. The coefficient of variation for quality control samples was less than ± 5%. The lower limit of quantification (LOQ) was 2 ng/ml, and the lower limit of detection (LOD) was 0.5 ng/ml.

### Pharmacokinetic analysis

The natural logarithm of quantified mefloquine concentrations was analysed to derive pharmacokinetic parameter estimates. A non-linear mixed-effect modelling approach was utilized using the NONMEM v7.3 software [19]. Various supporting software were applied for graphics diagnostics and facilitation of modelling. The R v3.2.0 (the R Foundation for Statistical Computing, Vienna, Austria) and the package Xpose v4.6.1 were used for graphical diagnostics, while Pirana v2.9.2 and Perl-speaks-NONENMEM v4.6.0 were used to facilitate the modelling process [19–23].

Individual parameter estimates were assumed to be log-normally distributed by adding between-subject variability with an exponential model according to Eq. (1) and the structural model best describing the mefloquine concentration data were evaluated.

1 $$P_{i} = \theta_{P} \cdot e^{{\eta_{i,P} }}$$

where P_i_ is the individual parameter estimate, θ_P_ is the typical value of parameter P, and η_i,P_ is the inter-individual variability for parameter P and patient is was drawn from a normal distribution with mean 0 and variance ω^2^.

One-, two-, and three-compartment disposition modelswere evaluated to describe the absorption phase. A first-order absorption, a first-order absorption with the lag-time model, and a transit compartment model (1–13 transit compartments) with the absorption rate and rates between transit compartments were either assumed to be equal or estimated separately [24]. Between dose–occasion variability was investigated on absorption parameters according to Eq. (2):

2 $$P_{i,O} = \theta_{P} \cdot e^{{\eta_{i,P} + \kappa_{O,P} }}$$

where P_i,occ_ is the individual parameter estimate at occasion O, and κ_O,P_ is the between-occasion variability of parameter P at occasion O drawn from a normal distribution with mean 0 and variance Π^2^.

The model was parameterized as follows: apparent elimination clearance (CL/F), central volume of distribution (Vc/F), inter-compartmental clearance(s) (Q/F), peripheral volume of distribution(s) (Vp/F), and mean transit time of the absorption phase (MTT).

Body weight was evaluated as an allometric function on all clearance and volume of distribution parameters. The exponents were fixed to 0.75 for clearance parameters and 1 for volume parameters based on previous reports [25, 26]. Other available covariates (e.g., body weight as a linear function, gender, and admission parasitaemia) were evaluated in a step-wise covariate search. In the forward step, the covariates were included with a statistical significance level of 0.01. The backward step was more stringent with a significance level of 0.001. In addition to the previously described covariates, validated molecular markers for mefloquine resistance and in addition, artemisinin resistance, were evaluated as covariates in the pharmacokinetic model.

Discrimination between two hierarchical models was based on the difference in objective function value (OFV), which is proportional to − 2 times the log-likelihood. The OFV was assumed to be Chi squared distributed resulting in a drop in OFV of 3.84 and 10.8 to be significant at statistical significance levels of 0.05 and 0.001, respectively.

Model fit was also evaluated using goodness-of-fit plots by plotting observations against the population and individually predicted concentrations and conditionally weighted residuals against population predicted concentrations and time after dose [27]. The performance of the model was evaluated with a visual predictive check [28], and shrinkage was calculated to evaluate individual estimates [29]. Parameter precision was calculated based on the result of 1000 resampled bootstrap datasets.

## Results

### Pharmacokinetic model

Population pharmacokinetic analysis of mefloquine was performed in 129 Burmese patients with uncomplicated falciparum malaria with a total of 653 data points. Thirty-six patients had the re-appearance of parasitaemia during days 21–40 of the 42-day follow-up period, and 93 patients had the sensitive response. Patient demographic data are summarized in Table 1. Baseline mefloquine concentrations were present in 5 patients (3.88%), and these measurements were ignored. Whole blood mefloquine concentration–time profiles were described by a two-compartment disposition model, which was superior to a one-compartment disposition model (*p *< 0.05). Adding on one additional disposition model was not significant (*p *> 0.05). The absorption phase was described by 1 transit compartments; this absorption model was superior to the other tested absorption models (*p *< 0.05). Estimating the transit rate constant and the absorption rate constant separately resulted in a significantly improved model (*p *< 0.01). However, it also resulted in a model with a low precision in the estimation of the apparent volume of distribution of the central compartment (a relative standard error of 46.9% based on 300 bootstrap runs). The addition of relative bioavailability (F) fixed to 100% with an estimated inter-individual variability did not significantly improve the model and was excluded from the final model (*p *< 0.05). The addition of allometrically scaled body weight (exponent fixed to 0.75 and 1) did not improve the model and was not kept in the final model. Finally, the addition of between-occasion variability on the absorption parameters did not improve the model and was therefore excluded from the final model.

**Table 1:** Demographics of the study population

	Median	Range (min–max)
Body weight (kg)	52.5	39–73.5
Age (years)	25	16–50
Sex (% females)	49.6	–
Admission parasitaemia (/µl)	5320	1260–84,000
PCT (h)	26	14–48
FCT (h)	26	18–42

*PCT is the parasite clearance time, FCT is the fever clearance time*

Covariates were investigated in a step-wise manner using the SCM functionality in Perl-Speaks-NONMEM. Of the covariates investigated (body weight, gender, and admission parasitaemia), none was found significant. Of the investigated molecular markers of mefloquine resistance and *kelch*-*13* mutations, only the *pfmdr1*-D12467 was found to have a significant impact on the pharmacokinetics of mefloquine. However, the increased relative bioavailability (before this parameter was excluded) found in patients with mutated parasites was deemed unlikely and was excluded from the final model. Significant differences in maximum concentration (C_max_) and elimination half-life (t_1/2_) were found in patients who had treatment failure (36 cases) compared to patients with successful treatment (107 cases).

**Fig. 1:**
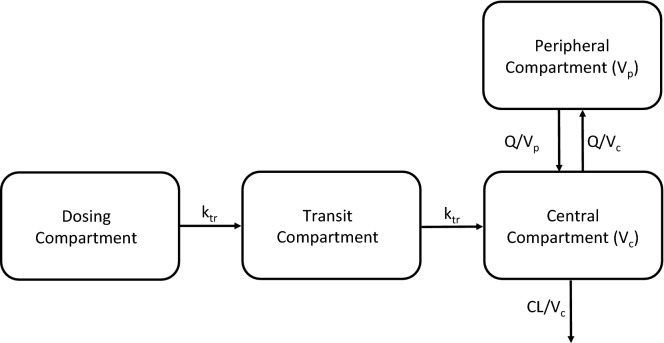
Graphical representation of the final model describing the pharmacokinetics of mefloquine. k_tr_ is the rate constant between absorption compartments, CL is the apparent elimination clearance, V_c_ is the apparent volume of distribution of the central compartment, Q is the inter-compartmental clearance between the central and peripheral compartment, and V_p_ is the apparent volume of distribution of the peripheral compartment

**Fig. 2:**
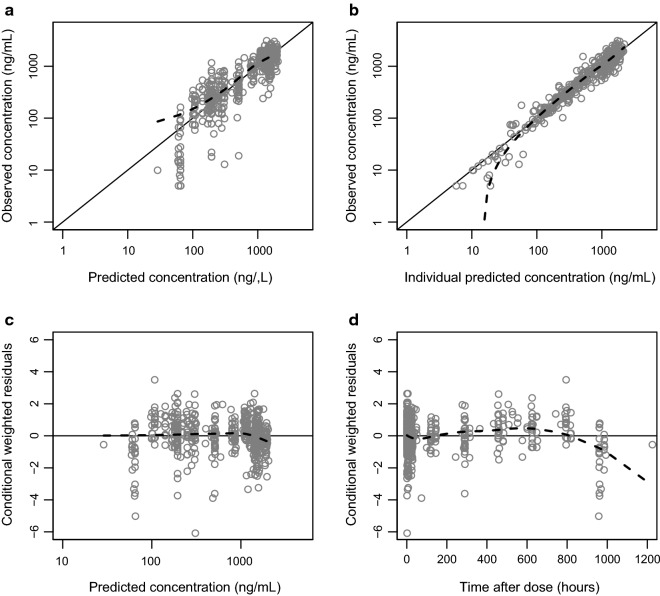
Goodness-of-fit of the final mefloquine model. Top left: observed concentration against population concentrations. Top right: observed concentrations against individually predicted concentrations. Bottom left: the conditional weighted residual against population prediction. Bottom right: the conditional weighted residual against time after the dose

**Fig. 3:**
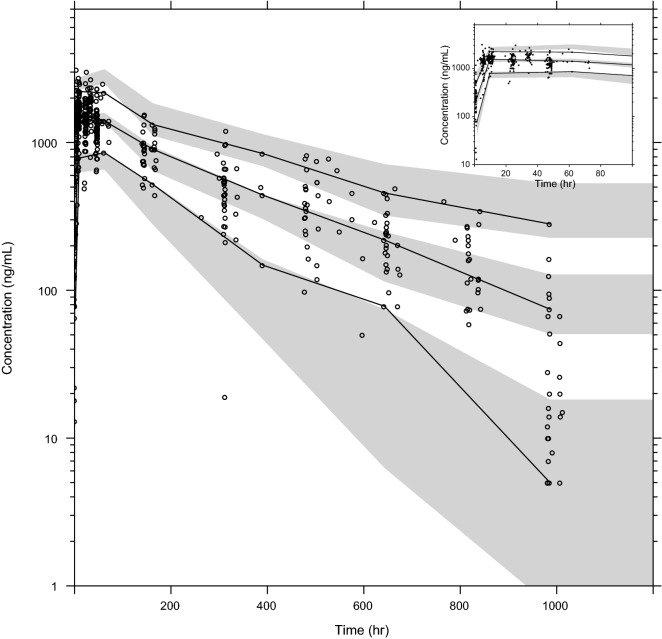
The visual predictive check of the final mefloquine model. Grey circles represent the observed concentrations. The solid black line represents the 50th percentile (median) of the observed data; the dashed lines represent the 5th and 95th percentiles, respectively. The shaded area represents the 95% confidence interval around the simulated 5th, 50th, and 95th percentile. The inset shows the absorption phase, between 0 and 100 h

A graphical representation of the final model is presented in Fig. 1. The models exhibited good predictive performance as can be seen in the goodness-of-fit plots and the visual predictive check (Figs. 2 and 3). A small trend was found in the residual plots for the late time point, probably due to the censoring of the data (lower limit of quantification). The visual predictive check indicated that this model exhibits good predictive performance. The parameters estimates, shrinkage, and parameter precision are presented in Table 2. Secondary parameters, i.e., C_max_, time to maximum concentration (t_max_), area under the concentration–time curve (AUC), and t_1/2_ are presented in Table 3.

**Table 2:** Parameter estimates from the pharmacokinetic model describing mefloquine

	Parameter value (%RSE)^ab^	95% CI^b^	IIV %CV (%RSE)^ab^	95% CI^b^	Shrinkage (%)^a^
CL/F (l/h)	2.77 (4.81)	2.52–3.04	38.0 (14.8)	27.1–48.0	33.7
Vc/F (l)	359 (3.38)	335–384	–	–	–
MTT (h)	3.89 (5.18)	3.54–4.32	43.6 (11.5)	33.7–53.0	18.5
Q/F (l/h)	11.7 (8.37)	10.1–13.9	55 (27.3)	22.4–80.5	50.8
Vp/F (l)	474 (8.02)	406–554	63.1 (15.5)	43.9–81.7	43.2
σ^2^	0.0902 (18.7)	0.0591–0.124	–	–	17.9

σ^2^ is the variance of the residual error. %RSE is the relative standard error calculated as 100 × median/(standard error). CV% is calculated as 100 × SQRT (EXP(ω^2^) − 1). The 2.5th and 97.5th percentiles are used to present the 95% CI (95% confidence interval)

CL/F, apparent elimination clearance; Vc/F, apparent volume of distribution of the central compartment; MTT, mean transit time of the absorption; Q/F, apparent inter-compartmental clearance; Vp/F, apparent volume of distribution of the peripheral compartment; F, relative bioavailability

^a^From NONMEM

^b^From bootstrap run with 958 successful runs (out of 1000)

**Table 3:** Secondary parameters for mefloquine

	Cured infection median (min–max) (n = 93)	Recrudescent infection median (min–max) (n = 36)	*p*-values
C_max_ (ng/ml)	2050 (1290–2410)	1980 (1280–2370)	0.0107
T_max_ (h)	7.54 (3.76–18.2)	7.50 (4.92–16.6)	0.832
AUC_0–150days_ (µg*h/ml)	450 (144–731)	455 (227–831)	0.554
t_1/2_ (days)	9.72 (1.97–16.7)	10.3 (5.58–17.9)	0.0449

C_max_, maximum concentration; T_max_, time to reach the maximum concentration; AUC_0–150days_, area under the concentration–time curve from the first dose to day 150 after the first dose; t_1/2_, terminal elimination half-life. P-values were calculated using an unpaired t test

## Discussion

Mefloquine has had a history of resistance development, but has been successfully used in Thailand in combination with artesunate [30, 31]. With the emergence of artemisinin and piperaquine resistance, mefloquine is reconsidered to be used in triple combination or as a substitute for dihydroartemisinin–piperaquine. It is therefore of importance to have access to population pharmacokinetic models for mefloquine describing the change in concentration over time [32, 33]. In a previous report on the same clinical study as the present analysis [9], low mefloquine exposure was shown to contribute to treatment failure following a 3-day artesunate–mefloquine combination. In this study, 7 cases with reduced sensitivity to mefloquine in combination with the changes in pharmacokinetics of either mefloquine and/or artesunate were identified. Two of these patients had resistance to mefloquine alone, while 5 had resistance to mefloquine combined with reduced sensitivity to the artemisinin derivative. Pharmacokinetic factor alone contributed to recrudescence in 3 cases, all of which had inadequate whole blood mefloquine levels during the first 7 days of treatment (AUC_0–7days_). Drug concentrations capable of killing the parasite (above 500 μg/l) must be achieved for at least 3–4 parasite multiplication cycles (7 days) to be confident that cure will be achieved [34]. In this study, it was not possible, due to insufficient power, to identify covariates that contribute to the variability in the pharmacokinetics of mefloquine. To investigate this further, a more detailed population pharmacokinetic approach was used. The current method provides estimates of mefloquine pharmacokinetic parameters and the variability between patients.

The present study describes the pharmacokinetics of mefloquine with individual parameter estimates using a non-linear mixed effect model. The pharmacokinetics of mefloquine was well described with a two-compartment model. This is in agreement with that previously reported [35–38]. Although some previously published models applied a one-compartment model to describe the concentrations of mefloquine over time [39–41], the present model resulted in a median mefloquine elimination half-life of 9.79 days, which is in agreement with previous studies. The different choices of distribution model are probably due to different study design and different amount of data in the elimination phase.

The mefloquine dose of 25 mg/kg of body weight was split into a 15 mg/kg dose followed by 10 mg/kg body weight dose to improve oral bioavailability and tolerability [42]. In the current study, mefloquine was well absorbed using this split-dose regimen. The absorption phase in the present model was described by one-transit compartment which successfully described the absorption phase. Previous models for mefloquine have primarily applied a first-order absorption model. However, the one-transit compartment has previously been identified in one study [36]. A transit-compartment model describes the observed lag-time in the absorption with the physiologically plausible model. Between dose and occasion variability was evaluated for absorption parameters to evaluate within individual variability and also to see if any trend in changing parameter estimates over time could be found. This is, for example, the case for piperaquine in which the relative bioavailability increases at later dose occasion [43, 44]. In the present study, this was not the case for mefloquine, and the absorption can be assumed to remain constant while the patients recovered from the disease.

Between-occasion variability has previously been identified on elimination clearance of mefloquine [38]. However, the study was performed over 6 months with 3 separate dosing occasions. The present study was performed over a 3-day period, and it was deemed highly unlikely that elimination clearance would change over the 3 doses. Therefore between-occasion variability on elimination clearance was not evaluated.

Parameter estimates of mefloquine population pharmacokinetics were similar to that previously published, and all parameters could be estimated with reasonable precision, indicating a stable model [37–42]. Shrinkage was low for all parameters except the inter-compartmental clearance, which was relatively high (50.8%). A trend could be seen in the conditional weighted residuals versus time after dose plot at late time points; this trend is a result of the last samples points which were quite low in this study.

Several covariates were investigated in the model. Body weight with fixed allometric exponents was investigated but was not found significant. The pharmacokinetics of several anti-malarial drugs, including piperaquine are affected by the body weight of the patients [43]. For mefloquine, some studies have identified an impact on body weight [34–37, 40]. In the present study however, no children were included, which explains why body weight was not identified as a significant covariate. Additional clinical studies including children of all ages would be useful to fully determine the impact of body weight on the pharmacokinetics of mefloquine. In addition to body weight, previous studies identified an effect of haematocrit and history of fever as covariates on the elimination clearance. Also, body temperature, haematocrit level, history of fever, the presence of malaria infection, and mixed species malaria infection were identified as significant covariates for the volume of distribution [34, 36, 40]. In the present study, information on haematocrit level and body temperature were not available for analysis and were therefore not included in the covariate search. All participants included in the study already had mono-infection with *P. falciparum,* and the impact of mixed infection was not evaluated. Admission parasitaemia was however, evaluated on all parameters and was not found significant. Validated molecular markers for mefloquine resistance were investigated to evaluate if any of those would affect the pharmacokinetic properties of mefloquine. The only marker retained after the backward step was *pfmdr 1*-D12467, estimating an increased relative bioavailability in mutated patients. This was deemed as an unlikely scenario from a biological standpoint and was not included in the final model. In addition, the analysis showed that mutation of the Kelch-13 propeller did not affect the pharmacokinetic parameters of mefloquine. The significant influence of artesunate administration on mefloquine pharmacokinetics is unlikely [45, 46] and was not included in the analysis.

The model was also used to derive the secondary parameters, identifying a significant difference in maximum concentration and elimination half-life between patients who had treatment failure and patients with successful therapy. This could indicate a different volume of distribution in these two groups. However, none of the available covariates successfully described this difference and additional more extensive studies are needed to evaluate why this difference was seen.

## Conclusion

This study successfully describes the pharmacokinetics of mefloquine following a 3-day artesunate–mefloquine in patients with uncomplicated falciparum malaria from Thailand. A model has been developed which adequately describes the pharmacokinetics of mefloquine. The developed model supports the previously published two-compartment models and a one-transit compartment absorption model. The differences in the maximum concentration and elimination half-life were also identified between patients with successful treatment and those with recrudescence. More extensive clinical studies including both adults and children are needed to fully characterize the pharmacokinetics of mefloquine.
